# Methods for Establishment and Maintenance of Germ-Free Rat Models

**DOI:** 10.3389/fmicb.2020.01148

**Published:** 2020-05-29

**Authors:** Lingling Qv, Zhenggang Yang, Mingfei Yao, Sunbing Mao, Yongjun Li, Jia Zhang, Lanjuan Li

**Affiliations:** State Key Laboratory for Diagnosis and Treatment of Infectious Diseases, National Clinical Research Center for Infectious Diseases, National Medical Center for Infectious Disease, Collaborative Innovation Center for Diagnosis and Treatment of Infectious Diseases, The First Affiliated Hospital, College of Medicine, Zhejiang University, Hangzhou, China

**Keywords:** gut microbiota, host–microbiota interactions, germ-free (GF) rodent, *in vivo*, protocol, establishment and maintenance

## Abstract

Numerous studies have demonstrated that the gut microbiota plays a vital role in human health and disease development. Although the number of studies on host–microbiota interactions have increased in recent years, the underlying pathogenesis of dysbiosis-related diseases are still largely unknown. Germ-free (GF) rodent models, with the animals housed in sterile isolators and completely free of microbiota, are useful tools to advance our understanding of host–microbiota relationship *in vivo*. Although protocols concerning the establishment and maintenance of GF mouse models have previously been reported, the establishment, maintenance and monitoring of GF rodents are labor-intensive, tedious and take experience and skills. The aim of our study was to establish a GF rat model for the following microbiota-related researches and provide an easy-to-use protocol for the establishment and maintenance of GF rat model in detail, including steps to set up the isolator, sterilize the flexible isolator bubble, import food, water and other supplies, and methods to acquire newborn GF rats, hand rearing of suckling GF rats and reproduction of GF offspring. During the hand feeding period, the body weight of suckling GF rats was weighed once a day to ascertain the amount of artificial milk was given. Based on our results, the body weight of suckling GF rats decreased 1 week after birth and then began to increase. Methods for verifying the quality of the model like assessing the sterile status of the rat colony are also described. Moreover, possible difficulties and challenges, especially during gavage, and suggestions to avoid contamination will be discussed. The protocol presented will facilitate the establishment of GF rat models and downstream microbiota-related researches.

## Introduction

The human gastrointestinal (GI) tract contains trillions of microbes, including bacteria, viruses, archaea, fungi, parasites and protozoa (Qin et al., 2010; Carding et al., 2017). Accumulating evidence suggests that the gut microbiota play a critical role in promoting human health through various means including production of vitamins, absorption of nutrients, pathogen displacement and development of the immune system (O’Hara and Shanahan, 2006). It was believed that the GI tract was sterile *in utero* and that the first colonization and initiation of gut microbiota began during the delivery process (Salazar et al., 2014). The structure and composition of the gut microbiota stay in homeostasis under normal conditions, and they can be altered by various factors, such as diet, age, and disease (Cresci and Bawden, 2015). Additionally, there was an increasing body of evidence showing that dysbiosis of the microbiota was associated with the pathogenesis of obesity (Gerard, 2016), diabetes (Munoz-Garach et al., 2016), cardiovascular diseases (Tang et al., 2017), inflammatory bowel disorder (Nishida et al., 2018), asthma (Kang et al., 2017), and allergies (Musso et al., 2019). However, the direct role of the gut microbiota in disease development and the underlying mechanisms relating dysbiosis to deteriorating health have not yet been fully elucidated and further researches on the topic are urgently needed.

Experimental models utilizing germ-free (GF) animals are invaluable tools for understanding how the gut microbiota may affect host physiology and metabolism, or for exploring interactions between the host and its microbiota (Grover and Kashyap, 2014; Bhattarai and Kashyap, 2016; Fiebiger et al., 2016; Kubelkova et al., 2016; Kennedy et al., 2018; Uzbay, 2019). The term “GF” refers to an animal being completely devoid of microbes, including bacteria, viruses, fungi, parasites, and protozoa, throughout its lifetime (Wostmann, 1996; Yi and Li, 2012). GF animals selectively colonized with one or more bacterial strains are referred to as gnotobiotic animals (Fritz et al., 2013). However, gnotobiotic sometimes was used as a synonym for GF (Gordon and Pesti, 1971; Umesaki, 2014; Kubelkova et al., 2016). The concept of GF animals was first proposed by Pasteur (1885), although he believed that surviving without bacteria was impossible. The first GF animal (a guinea pig) was generated by Nuttall and Thierfelder (1896) and managed to survive for 13 days. Because of the lack of knowledge concerning appropriate nutrition and adequate equipment, it was not until 1946 that the first GF rat colony was established (Gustafsson, 1946). The second successful report was presented by Reyniers et al. (1946) in the same year. Subsequently, the first GF mouse colony was developed by Pleasants (1959) in the United States. Since those early models, advances in the knowledge regarding nutritional differences, intestinal morphology, intestinal epithelium characteristics, intestinal function, metabolic characteristics, and mucosal immunity (Wostmann, 1981; Al-Asmakh and Zadjali, 2015) have enabled the development of substantially improved GF animal models.

The main principle behind the establishment of GF animal models is the theory that the fetus in the uterus is sterile (Stinson et al., 2017). Therefore, through aseptic cesarean section, the entire uterus containing the fetus can be removed and transferred into a sterile isolator. The fetus is then delivered from the uterus in the isolator and the neonate can be reared by a GF foster mother or artificially fed with formula milk (Arvidsson et al., 2012). The early GF animals were housed in stainless steel isolators (Gustafsson, 1959a). However, the early stainless steel isolators were heavy, expensive and inflexible, and have been replaced by more flexible, plastic polyvinyl chloride (PVC) isolators (Arvidsson et al., 2012; Basic and Bleich, 2019).

Protocols for establishing GF mouse models have previously been published (Arvidsson et al., 2012; Lavin et al., 2018). However, there are still differences between rats and mice. The aim of our study was to establish the GF rat colony for the following gut microbiota-related researches and provide a more detailed protocol for establishing and maintaining a GF rat model, including how to set up and sterilize the facility, how to perform the cesarean section, how to hand feed the neonates, and how to assess the GF status of the model. We also present results from our establishment of the model and quality control data as validation of the protocol. The protocol presented will facilitate the establishment of GF rat models and downstream microbiota-related researches.

## Methods

**FIGURE 1:**
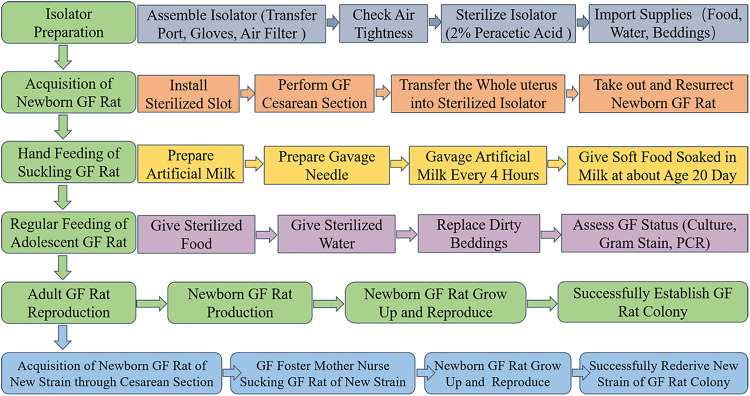
Schematic overview of the procedures involved in establishing GF rat model. Isolator preparation, acquisition of newborn GF rat, hand feeding of suckling GF rat, regular feeding of adolescent GF rat and reproduction of adult GF rat are the main steps. First, assemble the isolator accessories (including transfer port, gloves, and air filter), check air tightness, 2% peracetic acid to sterilize isolator and import supplies (including food, water, beddings and others that might needed) to make the isolator prepared. Second, install the sterilized slot, perform GF cesarean section, transfer the whole uterus into sterilized isolator, take out and resurrect newborn GF rat in the isolator to acquire newborn GF rat. Third, prepare artificial milk and gavage needle to hand feed the suckling GF rat through gavage. At about age 20 days, soft food soaked in artificial milk were given to the suckling GF rat instead of gavage. Fourth, sterilized food and water were given to the adolescent GF rat. Replace the dirty beddings and take fecal samples to assess the microbial status by culture, Gram-staining and PCR based on 16S rRNA gene. At last, adult male and female GF rat were caged together to reproduce GF offspring. The GF rat colony was successfully established when the population of GF rats remain stable. In addition, the new stain of GF rat colony can be established by sterile cesarean section and GF foster mother nursing.

A scheme summarizing the methodology for the establishment of a GF rat model is shown in Figure 1. In summary, the procedure consists of five main steps: isolator preparation, acquisition of neonate, hand feeding of the suckling rat, regular feeding of adolescent rat and reproduction of GF adult rat. In addition, the new strain of GF rat colony can be rederived by sterile cesarean section and foster nursing.

### Materials and Equipment

The GF rats should be kept in specialized isolators. A complete set of equipment for setting up the incubator was purchased from Suzhou Fengshi Laboratory Animal Equipment Co., Ltd. (FS-SPI-S, 1,400 × 800 × 1,600 mm, Suzhou, China), including the frame, air pump, removable metal air intake and outflow chambers (two per isolator), metal connector ring, flexible PVC bubble, plastic sheet (for covering the bottom of the bubble), internal and external port cover with rubber band or plugs, and other accessories such as PVC film, polypropylene (PP) filter paper, and stainless cylinder. The protocol for the assembly of the isolator will be described below.

A 2% solution of peracetic acid was used as a sterilizing agent for sterilization and maintenance of the housing environment (Thompson and Trexler, 1971; Basic and Bleich, 2019). Because the peracetic acid is unstable, the peracetic acid solution was prepared by mixing equal amounts of liquid A (a mixture of acetic acid and sulfuric acid) and liquid B (hydrogen peroxide) (Habo, Shanghai, China) before use and letting the mixture stand for 24–48 h, and subsequently diluting to a concentration of 2%. Sterilization with the peracetic acid solution was performed by spraying the solution with an atomizer consisting of an air compressor, an air hose, a spray gun (W-71, Anest Iwata, Japan) and a container.

**FIGURE 2:**
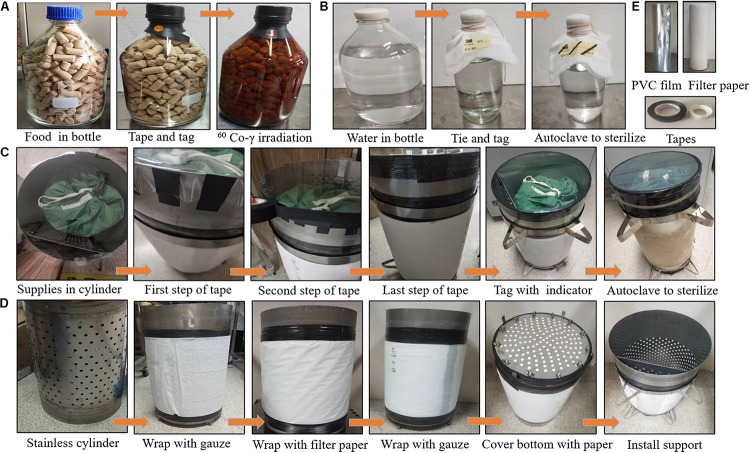
Methods for packing and sterilizing food **(A)**, water **(B)**, and beddings in the sterilizing cylinder **(C)**. Methods for manually packing the stainless cylinder **(D)**. PVC film, PP filter paper and tapes **(E)**. **(A)** Food was put in the bottle and the vinyl tape was used to seal the opening. The radiation indicator (P8104-10mm, GEX, United States) was tagged to monitor the irradiation quality. Then the packed food was sterilized with 50 KGy of ^60^Co-γ irradiation. **(B)** Water was put in the glass bottle, the gauze and cotton were used to seal the opening. The steam indicator tape (1322L, 3M, United States) was tagged to monitor the sterilization quality. Then the packaged water was autoclaved at 121°C for 1 h. **(C)** Beddings and other supplies that might needed were packed with cloth bag, then put into the sterilizing cylinder. The PVC film was used to cover the opening of the sterilizing cylinder and the vinyl and mylar tape were used to make a tight seal. Then the packaged sterilizing cylinder was autoclaved at 121°C for 1 h. The steam indicator tape was used to monitor the sterilization quality. **(D)** The sterilizing cylinder is made of the stainless cylinder wrapped with gauze and filter paper.

In addition, all food, water, beddings and other supplies which were put in the isolator were sterilized in advance. The packaging and sterilization methods for water, food, beddings and the sterilizing cylinder are shown in Figure 2. Briefly, food was put in glass bottles and irradiated with 50 kGy of ^60^Co-γ radiation (Radiation Center, Zhejiang Academy of Agricultural Sciences) (Figure 2A). Water was put in glass bottles and autoclaved for 1 hour (TOMY SX-500, Japan) (Figure 2B). Beddings in the cloth bag, gauze pieces, Eppendorf tubes (Eppendorf, Germany) in metal containers, and other heat-resistant supplies were put into the sterilizing cylinder, then the sterilizing cylinder was sealed with the PVC film and autoclaved at 121°C for 1 h to sterilize (Figure 2C). The sterilizing cylinder used for sterilization was manually packed as shown in Figure 2D. In brief, the stainless cylinder was wrap around with two layers of cotton gauze, five cycles of filter paper and two layers of cotton gauze step-by-step. And the vinyl and mylar tape were used to hold the cotton gauze and filter paper tightly on the cylinder. After that, the bottom of the cylinder was covered with fiver layers of filter paper and then secured with screws. Lastly, the supports were installed.

It is recommended to freshly prepare the artificial milk before use to decrease nutrient loss. The artificial milk used during gavage was prepared by using a simplified formulation based on a previously published formula (Jianguo Wang et al., 2007). The specific components and quantities of each ingredient are shown in Table 1. The rabbit milk, milk powder (Alfare, Nestle, Switzerland), fetal bovine serum (GIBCO, United States) and olive oil (Olivoila, Italy) were packed in each glass bottle separately and sealed with a gauze piece and cotton similar to the method for packing the water as described above, and then irradiated with 25 kGy of ^60^Co-γ radiation for sterilization. The water used to dissolve the milk powder was sterilized by autoclaving for 1 h. After sterilization, the components of the formula were imported into the isolator through the transfer port.

**TABLE 1:** The components of artificial milk (100 mL) used for feeding newborn GF rats at different ages.

**Components**	**Age of GF rat (days)**			
	**0–5**	**6–10**	**11–15**	**16–28**
Rabbit milk	52 mL	58 mL	60 mL	65 mL
Formula milk powder	3.7 g	3.9 g	3.6 g	3.8 g
Sterilized water	37 mL	26 mL	24 mL	19 mL
Fetal bovine serum	10 mL	15 mL	15 mL	15 mL
Olive oil	0	5 mL	5 mL	10 mL

Specific-pathogen-free (SPF) pregnant Sprague-Dawley (SD) rats were purchased from Zhejiang Academy of Medical Science. All the animal experiments were approved by the Laboratory Animal Ethics Committee of the First Affiliated Hospital of Medical college of Zhejiang University (permit number: 2017-400-3) and were performed in accordance with criteria of “Guide for the Care and Use of Laboratory Animals” (NIH publication).

### Isolator Preparation

**FIGURE 3:**
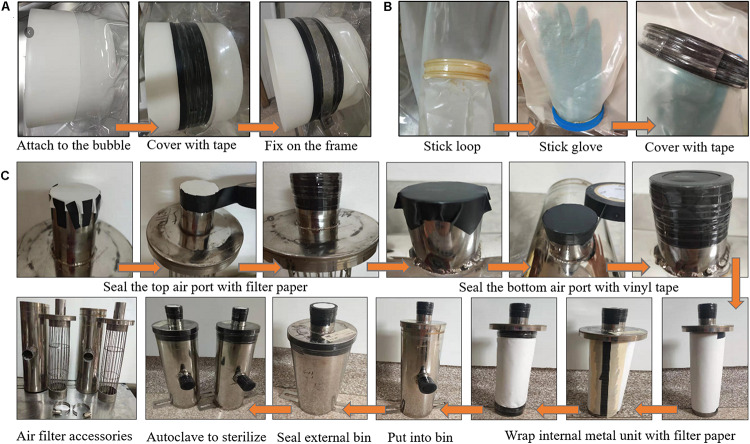
Methods for installing the transfer port **(A)** and gloves **(B)**, and for manually packing the air filter apparatus **(C)**. **(A)** The transfer port was attached to the isolator bubble, covered with vinyl and mylar tape, then fixed on the isolator frame. **(B)** The loop was covered with liquid glue, then trapped in the isolator bubble. The glove was stick on the loop with liquid glue. At last, the glove was covered with vinyl and mylar tape to make a tight seal. **(C)** The top and bottom air port of the air filter was sealed with filter paper and vinyl tape, respectively, by using vinyl and mylar tape. The inner metal unit was wrapped with filter paper four (air outflow chamber) to five (air intake chamber) times. Then the inner metal unit was put into the external bin and make a tight seal with vinyl and mylar tape. The packaged air filter was autoclaved at 121°C for 1 h and the steam indicator tape was used to monitor the sterilization quality.

**FIGURE 4:**
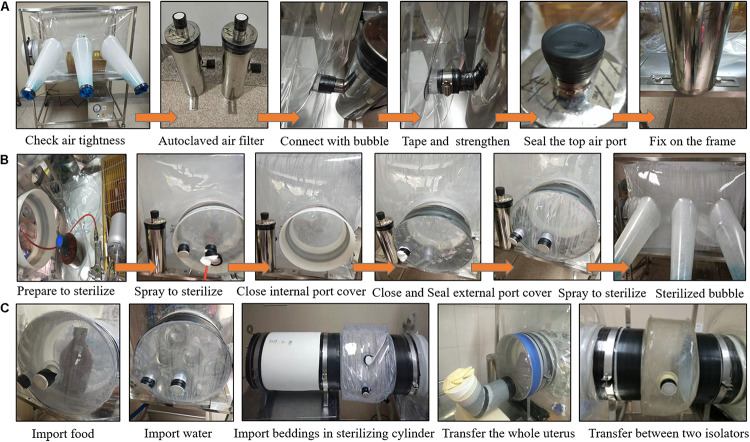
Methods for installing the air filter apparatus **(A)**, sterilizing the isolator bubble **(B)**, importing food, water, beddings and for transferring neonates and between different isolators **(C)**. **(A)** After the air tightness of the isolator bubble was checked, the autoclaved air filter was connected with the bubble through the air port on the isolator bubble, sealed with vinyl and mylar tape, strengthened with the metal connector ring, the top air port of the air filter was sealed with vinyl tape, then the air filter was fixed on the isolator frame with screws. **(B)** The atomizer container filled with 2% peracetic acid was placed in the isolator bubble, the external port cover was closed with the air horse passing through the port, then the sterilizing agent was sprayed unto the bubble. After spraying, the internal port cover was closed with the rubber band strengthened, the spray gun and container were taken out, then the external port cover was closed and sealed with vinyl tape. Finally, the transfer port was sterilized with 2% peracetic acid. **(C)** The sterilized food and water were imported into the isolator bubble trough the transfer port directly. The sleeve was used to import beddings and other supplies in the sterilizing cylinder and transfer between two isolators. The U-shape slot filled with 2% peracetic acid was used to transfer the whole uterus.

Preparation of the isolator entails isolator set up, sterilization and importation of previously sterilized water, food, beddings, and other supplies necessary for the rearing of the GF rats. Methods for assembling and sterilizing the isolator and how to import supplies into the isolator are shown in Figures 3, 4.

#### Methods for Assembling the Isolator

The first part of assembling the isolator was to install the transfer port (Figure 3A). The transfer port was attached to the isolator bubble and taped tightly with vinyl and mylar tape step-by-step, then fixed on the isolator frame with screws. It should be noted that if the cage cannot pass through the transfer port directly, the cage should be put in the isolator bubble before the installation of the transfer port.

Secondly, the gloves were installed (Figure 3B). The gloves are the media through which to operate inside the isolator without direct contact between the outside environment and the animals inside. Liquid glue was used to stick the loops on the sleeves of the isolator bubble. Then, some liquid glue was laid over the loops and the gloves were put inside and attached on the loops. The gloves were taped with vinyl and mylar tape to ensure that the gloves were closely attached on the loops. After that, with the internal and external port cover covered, the flexible bubble was inflated by air pumped through the air intake port. After inflation of the bubble, the air intake and outflow port were plugged with plugs to check the air tightness of the gloves and the transfer port.

Lastly, the air filter apparatus was installed (Figure 3C). The air intake and outflow chamber consist of an internal metal filtration unit and an external bin. Filter papers were wrapped around the air outflow and intake metal unit four to five times, respectively. The papers on the metal unit were held with vinyl and mylar tape. Then, the filtration units were put into the external bin and sealed with vinyl and mylar tape. The mylar tapes were stretched over the vinyl tape to form a tight seal. Moreover, the top air port of each metal unit was sealed with filter papers and the bottom air port of the external bin was sealed with vinyl tapes. The air intake and outflow chamber were subsequently sterilized by autoclaving for 1 h. Steam indicator tapes (1322L, 3M, United States) were used to monitor the sterilization quality. Then, the sterilized air intake and outflow chamber were connected with the flexible bubble through the air intake and outflow port. The metal connector rings were used to make sure they are tightly connected. Finally, the air filtration apparatus was attached to the isolator frame with screws (Figure 4A).

#### Methods for Sterilization of the Isolator Bubble

After ensuring that the isolator was air tight, that the autoclaved air intake and outflow chamber were connected to the bubble and fixed on the isolator frame and that the top air port of the air filter apparatus was sealed with vinyl tape, the bubble was sterilized (Figure 4B). First, 1,000 mL of 2% peracetic acid solution was prepared and placed in the atomizer container inside the bubble. The external port cover was closed with the air hose passing through the port, and then the air hose was connected to the spray gun, which in turn was connected to the container inside the bubble with the other side of the air hose connected to the air compressor. Then, the peracetic acid solution was sprayed unto the interior surfaces of the bubble. After application of the sterilizing agent, the bubble became foggy from the droplets of the peracetic acid solution and the spray gun and container were put in the transfer port and the internal port cover was closed and strengthened with rubber band. Lastly, the external port cover was closed and vinyl tape was applied to make a tight seal with the transfer port. The sterilizing agent was sprayed unto the interior surface of the transfer port through the port on the external cover, and then the port of the external cover was plugged with plugs. The vinyl tape was used to seal the plugs to make sure no air could enter into the transfer port.

#### Methods for Importing Supplies Into the Isolator

To facilitate work on experiments in the isolator, food, water, beddings, and other supplies should be imported into the isolator bubble in advance. As shown in Figure 4C, the packed and sterilized food and water were transferred into the bubble through the transfer port directly by opening the external port cover, the water and food were put inside, the cover was closed and sealed with vinyl tape, then the inside of the transfer port was sterilized by spraying the sterilizing agent through the port on the external cover. After sterilization, the plugs were used to block the port of the external port cover and sealed with vinyl tape to ensure that no air could get inside. A total of 2 h after the sterilization procedure, the rubber band was taken off, the internal port cover was opened, and water and food were taken out and transferred into the isolator bubble. Subsequently, the internal port cover was closed and strengthened with rubber band.

Unlike water and food, supplies were imported through the sterilizing cylinder into the isolator bubble through a plastic sleeve. Firstly, the sterilizing cylinder and transfer port were connected with a plastic sleeve, the vinyl tape was used to seal it tightly, the sterilizing agent was sprayed through the port on the sleeve, then the port was blocked with plugs and the plugs were sealed with vinyl tape. A total of 2 h after sterilization, the rubber band was taken off, the internal port cover was opened, the PVC film was punctured (e.g., with a scissor), the beddings and other supplies in the cylinder were taken out and transferred into the isolator bubble. Lastly, the internal port cover was closed and strengthened with the rubber band, the vinyl tape was torn off, the cylinder and sleeve were removed, then the external port cover was closed and the transfer port was sterilized as described above.

To prevent contamination, every time the external port cover was open, the internal port cover should be closed, and the transfer port should be sterilized with the sterilizing agent. Additionally, the empty bottles and dirty beddings should be transferred out through the transfer port from time to time to keep the interior of the isolator bubble orderly.

### Acquisition of Newborn GF Rats Through Cesarean Section

Newborn GF rats can be acquired through sterile cesarean section by taking out the whole uterus based on the theory that the GI tract is sterile *in utero* (Salazar et al., 2014). Although this theory has recently been challenged (Hou et al., 2013; Mandar et al., 2015; Verstraelen et al., 2016), hysterectomy was still the most important method for obtaining sterile animals. In our experiments, only one pregnant SD rat was used for cesarean section to obtain GF newborns in each experiment. Test 1 was conducted in June 2017, test 2 was conducted in August 2017 and test 3 was conducted in February 2018, therefore three pregnant SD rats in total were sacrificed for the acquisition of GF neonates. The specific steps of this procedure are described below.

Firstly, a pregnant SD rat in labor was euthanized by the manual cervical dislocation method (Carbone et al., 2012) and completely immersed in 2% peracetic acid which had been preheated to 37°C for 10 s. Secondly, the pregnant rat was secured on its back to reveal the entirety of its abdomen. The whole abdomen was sterilized by application of a cotton ball soaked in 2% peracetic acid. Thirdly, the skin of the abdomen was cut along the mid-abdominal line with sterilized surgical scissors to fully expose the uterus, the junction of the cervix and vagina was clamped with clamps and cut off with sterilized scissors from the opposite side of the uterus, then the entire uterus was carefully removed. Lastly, the uterus was sterilized by complete immersion in 2% peracetic acid (preheated to 37°C) for 5 s and subsequently put into the U-shaped slot, which had been filled with 2% peracetic acid and connected with the sterilized isolator in advance (Figure 4C).

The opening of the slot was covered with a rubber glove and the inner port cover was opened after 15 s. The whole uterus was then pulled out and transferred into the isolator through the installed gloves. Then, the internal port cover was closed with rubber band strengthened. Immediately afterward, the whole uterus was rinsed with distilled water and the neonates were removed from the uterus in the isolator bubble by using the installed gloves. The amniotic fluid and mucus on the bodies of the neonates, especially on the nose and mouth, was cleaned up. This procedure also helps to stimulate respiration. The umbilical cord was severed after 2 h. Additionally, the slot was removed and the external port cover was closed and the transfer port was sterilized with the sterilizing agent after the neonates were transferred into the isolator bubble.

### Artificial Feeding of the Newborn GF Rat by Gavage Until Weaning

**FIGURE 5:**
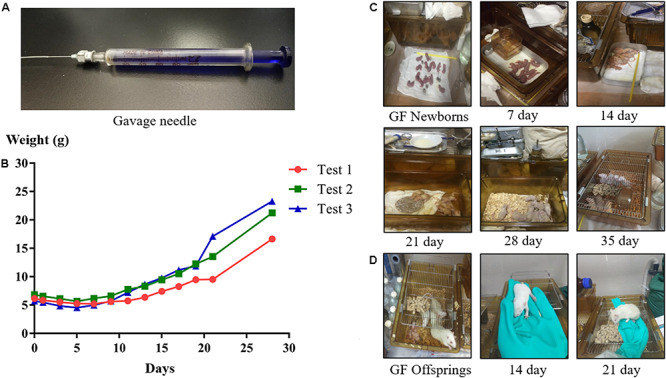
The gavage needle **(A)**. Growth curves **(B)** and photos of suckling GF rats **(C)** during the hand feeding period. Photos of GF offspring rats **(D)** at different ages.

The newborn GF rat obtained through aseptic cesarean section (as described in the previous section) must be kept in the sterile isolator to avoid contamination. Since they were not capable of sucking milk by themselves, the newborn GF rats were hand fed by gavage until weaning. Due to the fragility of neonates, the metallic gavage needle was inappropriate. A micro medical tubing (inner diameter 0.28 mm, outer diameter 0.64 mm, SCI, United States) was connected to the needle and then to the 1 mL syringe with calibration (Figure 5A), which was used as the tool for the gavage. The specific method of gavage is described below.

The rat’s head was held, and the rat’s body was kept upright, then the tube was inserted slowly through the mouth into the stomach, and the artificial milk was injected. In general, the suckling GF rats were fed every 4 h, six times a day, and the equal dose of milk was given each time. The amount of milk they drink in one day is ∼20% of its body weight (e.g., a rat weighing 6 g should be given 1.2 mL milk a day). To know what amount of milk to give to the rats, the rats were weighed under fast once a day.

During gavage, made sure that there was no air flow into the stomach of the rat. The times of gavage and the amount of milk fed to the rat were increased or decreased according to the digestive status of rat. Since suckling rats are hairless, it is possible to discern whether there is milk in the stomach or not by visible inspection. Moreover, it is important to stimulate suckling rats to defecate and urinate by using a cotton swab. The temperature and humidity in the isolator during the feeding process should be kept at 31–33°C and 50–65%, respectively. When the suckling rats’ eyes were completely open and their teeth were long enough, the gavage was stopped and the rats were instead given soft food soaked in milk to train their ability to eat by themselves. According to the different growth status of the suckling GF rats in each experiment, at which day to stop hand-rearing was different. In our experiments, the suckling GF rats began to eat soft food at day 22 in test 1, at day 19 in test 2, and at day 18 in test 3. When the rats could drink water by themselves, they were weaned and instead supplied with normal, solid food and water. Additionally, the temperature in the isolator was adjusted to about 26°C.

### Establishment and Rederivation of the GF Rat Colony

As mentioned above, the weaning GF rats should be kept in the sterilized isolator at all times. When the rats were old enough to eat and drink by themselves, sterilized food and water needs to be regularly provided, and the dirty beddings need to be replaced to maintain an orderly environment in the isolator. Male and female rats should be caged separately until they were old enough and ready to reproduce. In general, SD rats about 90 days old were of a suitable age to reproduce. To facilitate reproduction, put the male and female rat in the same cage and separate them when the female rat was certain to be pregnant. Abdominal palpation at about 14 days after mating was used to judge whether the rat was pregnant or not. After approximately 21 days of gestation, the pregnant female rat will give birth to GF neonates. When the newborn GF rats grow up and can reproduce, the population of GF rats can remain stable, the GF rat colony has been successfully established.

Additionally, the female rat which has just given birth can be used as a GF foster mother to nurse the new strains of newborn GF rats (acquired through sterile cesarean section, as described above) until they can eat and drink by themselves. As the new strain of newborn GF rats grew up and can reproduce, the new strain of GF rat colony was successfully rederivated. It should be noted that foster nursing can only be applied to closely related strains of rats.

### Assessment of GF Status by Analyzing Presence of Fecal Contaminants

Assessment of the GF status is an essential aspect in establishing and maintaining a GF rat model. Indeed, cecum enlargement is the most striking anomaly in GF rodents. This phenomenon is due to the accumulation of mucus, a mixture of macromolecular, sulfate-containing glycoproteins that normally are degraded by the microflora of the lower gut (Wostmann et al., 1973; Wostmann, 1981). However, fecal microbe-based detection is the most recognized method for assessment of GF status.

Three typical methods for assessing the GF status were used for validation in the current study and are described in detail below. They consist of cultivation of stool samples under aerobic and anaerobic conditions, Gram staining of fecal specimen followed by light microscopy and PCR-based detection using universal primers targeting the bacterial 16S rRNA genes (Smith et al., 2007; Bhattarai and Kashyap, 2016; Lavin et al., 2018). However, most intestinal bacteria are obligate anaerobes and difficult to cultivate, and minor contaminations may not be observed in Gram stained preparations. Moreover, PCR can give a false positive result since the method can detect the DNA of dead bacteria. Given the limitations of these techniques, it is necessary to evaluate the results of all three methods.

#### Culturing of Stool Samples

Culturing is the most used method to detect whether there is fecal contamination of bacteria or not. The culture medium and specific culturing method were selected according to earlier studies (Gustafsson, 1959a; Fiebiger et al., 2016). Fecal pellets were collected in autoclaved Eppendorf tubes and then transferred out of the isolator and inoculated in brain heart infusion (BHI) broth, fluid thioglycollate (FT) medium, tryptic soy broth (TSB), and two blood agar plates, respectively. Then the BHI, FT, and TSB medium were cultured under aerobic conditions at 37°C for at least 7 days. One blood agar plate was cultured under aerobic conditions, and another one was cultured under anaerobic conditions (AnaeroPack, MGC, Japan) at 37°C for 2 days. SPF rat feces and sterile saline should be used as positive control and negative control, respectively, to ensure the accuracy of the results. After 7 days of cultivation, a clear culture medium indicates that the sample was sterile. The absence of bacterial colonies on the plates indicates an absence of contamination.

Both the results of the cultivation in broth and on agar should be evaluated. It is recommended to do these tests weekly. Importantly, not all bacteria are cultivable by these culturing methods, and so negative results does not conclusively prove that the samples are free from contamination. As such, it is important to complement cultivation methods with other methods to ascertain the sterility of the GF rat model.

#### Gram Staining of Fecal Specimen

Staining-based methods are convenient methods used to detect the presence of bacteria in samples. In addition to bacteria detectable by culturing, staining-based detection methods can also detect non-culturable bacteria. In contrast to culturing, staining methods also give information on size and morphology of the contaminant. Although various single step staining methods are available (e.g., staining with methylene blue), Gram staining is a particularly useful staining method since it additionally can provide broad bacterial typing information (i.e., Gram positive or Gram negative) on detected contaminants.

Gram staining was performed according to a previously published protocol (Claus, 1992). In short, the fecal sample was coated on the surface of sterile glass slides with subsequent fixing of the preparation with alcohol lamp heating after it dried naturally. The preparation was then dyed with ammonium oxalate crystal purple for 1 min, rinsed with distilled water, dyed with iodine solution for 1 min, rinsed once more with distilled water, decolorized with 95% ethanol for 30 s to 1 min, rinsed again with distilled water, and finally dyed with safranin for 1 min, and then washed a final time with distilled water. After the preparation was completely dry, a light microscope was used to determine the presence of bacteria. If there are bacteria in the sample, Gram-positive bacteria will be stained purple and Gram-negative bacteria will appear red. A shortcoming of the Gram staining method is that the detection limit is fairly high. Moreover, the method does not discriminate between living and dead bacterial cells which can lead to false positive results.

#### Detection of Bacterial Contamination by Using PCR

The bacterial 16S rRNA gene contains regions conserved through most eubacterial species. By using primers complementary and specific to these regions, bacteria can be detected with high sensitivity and specificity with PCR-based methodology. In the current study, DNA was extracted from the fecal samples by using a fecal DNA extraction kit (12830-50, QIAGEN, Germany). A PCR reaction mixture consisting of the extracted DNA as template, dNTPs and DNA polymerase (TAKARA, Japan) in a PCR reaction buffer with bacterial universal primers (27F, 1492R) targeting the 16S rRNA gene (Dymock et al., 1996), was put through various cycles of heating and cooling in a thermal cycler. The details of the 16S rRNA gene-based PCR protocol is presented in Table 2.

**TABLE 2:** Primer sequences, composition of the reaction mixture and thermal cycling protocol for a 16 rRNA gene-based PCR for detection of eubacterial contamination.

**Primer sequences**	
27F	5′-GTGCTGCAGAGAGTTTGATCCTGGCTCAG-3′
1492R	5′-CACGGATCCTACG GGTACCTTGTTACGACTT-3′
**Composition of the PCR reaction mixture**	
2× PCR Mix	12.5 μL
27F (10 μmol/L)	1 μL
1492R (10 μmol/L)	1 μL
Template DNA	1 μL
Add ddH_2_O to	9.5 μL
**Thermal cycling protocol**	
First step	95°C Denaturation 5 min
Second step (35 cycles)	95°C Denaturation 30 s
	52°C Annealing 30 s
	72°C Extension 2 min
Third step	72°C Extension 10 min

In addition to the template DNA, a positive control using DNA extracted from SPF rat feces as template DNA and a no template control using distilled water instead of template DNA were run simultaneously to the PCR for the GF rat feces template DNA to ensure the quality of the results. The PCR products were subsequently analyzed by using agarose (1%) gel electrophoresis. A notable shortcoming of this method is, however, that PCR will detect DNA from dead bacterial cells in addition to DNA from living cells, which can yield false positive results.

## Results

### The Overview of Newborn GF Rats and Weaned GF Rats

As described above, only one pregnant SD rat was euthanized for hysterectomy to acquire GF newborns in each experiment. In Test 1, 13 GF neonates (male/female: 7/6) were obtained; in Test 2, 11 GF neonates (male/female: 5/6) were obtained; and in Test 3, 10 GF neonates (male/female: 6/4) were acquired (Table 3). The body weight of newborn GF rats ranged from 5.2 to 7.3 g and very few weighed less than 5.0 g. Repeated measures ANOVA was used to analyze the significant differences among different tests. The body weight of newborn GF rats in Test 1 and Test 2 have significant differences compared to Test 3 (*P* < 0.05, *P* < 0.05, respectively), whereas there is no significant differences between Test 1 and Test 2 (*P* > 0.05). After the hand-feeding period, a total of 18 GF rats survived, nine of which were male and female rats. The body weight of weaned GF rats in Test 1 has significant differences compared to Test 2 and Test 3 (*P* < 0.05, *P* < 0.05, respectively), whereas there is no significant differences between Test 2 and Test 3 (*P* > 0.05) (Table 3).

**TABLE 3:** The overview of newborn GF rats and weaned GF rats.

	**Newborn GF rats**			**Weaned GF rats**			**Survival rate**
	**Total**	**M/F**	**Body weight**	**Total**	**M/F**	**Body weight**	
Test 1	13	7/6	6.4 ± 0.5^a,b^	6	3/3	16.7 ± 0.7	46.1%
Test 2	11	5/6	6.4 ± 0.9^a^	6	2/4	21.2 ± 1.1^c^	54.5%
Test 3	10	6/4	5.7 ± 0.4	6	4/2	23.3 ± 2.9^c,d^	60.0%

*M/F is short for Male/Female. Results of body weight are expressed as the mean ± SEM. ^*a*^*P* < 0.05 compared to Test 3. ^*b*^*P* > 0.05 compared to Test 2. ^*c*^*P* < 0.05 compared to Test 1. ^*d*^*P* > 0.05 compared to Test 2.*

### The Growth Status of GF Rats

Due to the time it needed to prepare sterile isolator bubble, food, water and other supplies, and about a month of artificial feeding cycle, we did the experiment almost once in 45 days. The results of three of the experiments (Test 1, Test 2, and Test 3) are shown below to illustrate the growth status of GF rats (Table 4 and Figure 5). Our results showed that the suckling rats’ ears were erected at about age 3 to 5 day and that the rats started to grow fur and teeth at about age 8 day. Their eyes were completely open at about age 16 day. In general, the body weight of the suckling GF rats decreased 1 week after birth and then began to increase (Figure 5B). Moreover, when they were able to eat soft food soaked in milk by themselves and stopped being reared, their body weight increased quickly. As shown in Table 4, the body weight at weaning were related to the time of starting to eat soft food by themselves, and the earlier they were eaten soft food, the heavier they were when they were weaned. At age 28 day, the body weight of rats in Test 3 were heavier than rats in Test 2 and Test 1, while the rats in Test 3 had lower birth weight than those rats in Test 2 and Test 1. In addition, our results showed that rats weighing less than 5 g at birth were unable to live until weaning. Generally, the suckling GF rats were able to eat soft food at about age 20 day and eat solid food and drink water at about age 28 day (Figure 5C).

**TABLE 4:** The body weight (g) of survived suckling GF rats (*n* = 6) at different ages (days).

**Days**	**0**	**1**	**3**	**5**	**7**	**9**	**11**
Test 1	6.2 ± 0.7	5.9 ± 0.6	5.5 ± 0.5	5.3 ± 0.3	5.2 ± 0.2	5.6 ± 0.4	5.7 ± 0.3
Test 2	6.8 ± 0.5	6.6 ± 0.6	6.1 ± 0.5	5.7 ± 0.5	6.2 ± 0.6	6.6 ± 0.6	7.8 ± 0.5
Test 3	5.7 ± 0.4	5.5 ± 0.4	4.9 ± 0.2	4.5 ± 0.2	5.0 ± 0.2	5.7 ± 0.5	7.2 ± 1.0

**Days**	**13**	**15**	**17**	**19**	**21**	**28**	

Test 1	6.4 ± 0.3	7.4 ± 0.3	8.3 ± 0.2	9.5 ± 0.5	9.5 ± 0.5	16.7 ± 0.7	
Test 2	8.3 ± 0.8	9.4 ± 0.7	10.5 ± 0.7	12.2 ± 1.2	13.5 ± 0.6	21.2 ± 1.1^a^	
Test 3	8.7 ± 1.0	9.8 ± 1.2	11.2 ± 1.3	11.9 ± 1.7	17.1 ± 3.1	23.3 ± 2.9^a,b^	

*Results are expressed as the mean ± SEM (*n* = 6). ^*a*^*P* < 0.05 compared to Test 1. ^*b*^*P* > 0.05 compared to Test 2.*

After weaning, the adolescent GF rats were given sterile food and water. Their body weight and shape were like normal rats. At about age 90 day, the female and male rats were caged together to facilitate reproduction. The offspring rats feeding naturally from their biological mother grew better than the rats hand fed through gavage. This results is consistent with previous reports (Luckey et al., 1955). The difference of body weight and the growth status of fur could be directly observed (Figure 5D).

### Assessment of Sterility

To ensure that immediate action can be taken in case of contamination, regular monitoring of the environment in the isolator is necessary and sterility tests were done weekly. Culturing, Gram staining and PCR were used to test the GF status of the rats. The fecal samples of the newborn rats acquired by hysterectomy were the first samples to be tested. After that, the stools of rats were collected once a week to assess the sterility. The BHI, FT, and TSB broths supplemented with fecal samples showed no visible growth of bacteria after 7 days of incubation, and the blood agar plates showed no growth of colonies after 2 days of cultivation. No visible bacterial cells could be discerned in the Gram stained preparation of the fecal sample under a light microscope. The PCR assay could not detect any 16S rRNA genes in the extracted DNA from the fecal samples as indicated by the absence of bands in the gel electrophoresis.

## Discussion

Germ-free rats are attractive for research use because they do not carry any microbes in their guts, therefore, well-suited to study host–microbial interactions in health and disease. Comparison of GF and conventional animals and colonizing of GF animals with commensal bacteria are the most effective methods to directly and indirectly prove correlations between microbiota and disease phenotypes (Li and Wei, 2019). However, establishment and maintenance of GF animals are not as easy as it looks like. According to our experimental results, acquisition, maintenance and quality control are the most important aspects in establishing a GF rat model. In addition, the possible difficulties and challenges for the establishment of GF rat colony and tips for the establishment of GF mouse colony will also be discussed below.

### Challenges for Acquisition of GF Rat Colony

Acquisition of GF rats entail generation of newborn GF rats, hand-rearing to acquire adolescent GF rats and subsequent GF offspring rats. The initial delivery of newborn GF rats through sterile cesarean section should be carefully performed to avoid contamination. In addition, the dogma that the fetus is sterile in the uterus, which is the basic principle in the establishment of GF animal models, has been challenged. Studies from the recent decade have indicated that diverse microbial communities may exist in human semen and in the womb (Hou et al., 2013; Mandar et al., 2015; Verstraelen et al., 2016). And yet, another study concluded that the human placenta does not have a microbiome (de Goffau et al., 2019). Therefore, more research is needed to elucidate this controversy.

**FIGURE 6:**
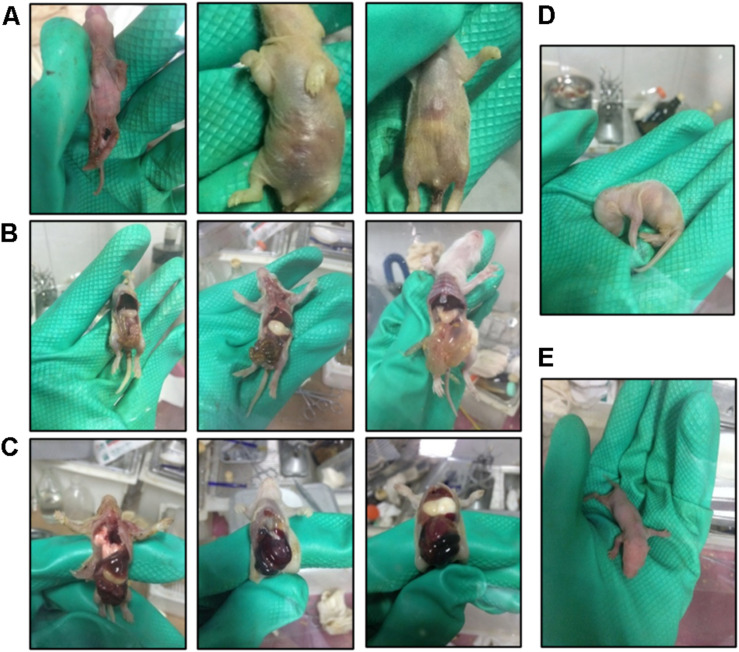
Photos of suckling GF rats during the hand feeding period. **(A)** Malnourished and diarrheal suckling GF rats. **(B)** Dead suckling GF rats with flatulence in the stomach and intestine. **(C)** Dead suckling rats with intestinal hemorrhage. **(D,E)** Suckling rats in different positions.

Hand-rearing of suckling GF rats is another challenging and critical step. Successful rearing is influenced by various factors, including the body weight of newborn GF rats, proficiency in gastric gavage and changes in temperature and humidity of the environment. During the periods in which artificial feeding is necessary, the neonates are small and fragile, and care should be taken during gavage to avoid inserting the gavage needle into the trachea. Furthermore, the pups have low immunity and the lack of microbes in their gut complicate digestion (Luczynski et al., 2016) of milk. For this reason, pups risk dying from flatulence in the stomach and intestine (Figure 6). Intestinal bleeding may occur in severe conditions. According to our experience, slight flatulence in the stomach can be relieved by reducing the amount of milk fed, extending the feeding interval or diluting the artificial milk with water. However, the prognosis of severe flatulence in the stomach or intestine and intestinal bleeding is not good. The mortality rate of intestinal bleeding is 100%, which is related to the deficiency of vitamin K in GF rats (Gustafsson, 1959b). Generally, if not treated promptly, slight flatulence in the stomach can develop into intestinal flatulence and bleeding, therefore it is important to pay attention to the digestion in the stomach. Additionally, temperatures below 31°C in the isolator can cause flatulence in the stomach and indigestion; an electric blanket underlay for the bottom of the isolator bubble can be used to keep the warmth.

Regarding adult rats, the low nutrition of repeatedly sterilized food and poor nutrient absorption of GF rats may contribute to infertility. For irradiation sterilization, we recommend the use of food with higher nutritional value.

### Challenges for Maintaining a Sterile Environment

After successful acquisition of GF rats, maintaining a sterile environment is a continuous challenge. Repeated importation of food, water and supplies, transfer of rats between different isolators and overall isolator management may lead to microbial contamination. Unlike many other forms of experiments, any mistake risks contaminating the entire experimental model, necessitating expensive and time-consuming re-establishment of a sterile GF rat colony. Hence, every step in establishing the model and all work performed in the isolator should be carefully performed and quality control is essential to carry out often. Furthermore, we recommend to regularly sterilize the isolator bubble and to replace the filter paper of the air filter apparatus and sterilizing cylinder. Additionally, the gloves are the most vulnerable parts, which should be inspected for any damage before each use to prevent possible contamination and changed before it is too late.

### Challenges for Quality Control of GF Rat Colony

As mentioned above, quality control of the GF status is essential; however, ascertaining the sterile status of the GI tract is difficult. The currently used methods to assess the quality of GF animals, aerobic and anaerobic culturing of fresh fecal samples, microscopic examination of stained fecal sample preparations and PCR methods based on the 16S rRNA gene (Smith et al., 2007; Bhattarai and Kashyap, 2016; Francois et al., 2016) all have their limitations. In addition, viruses do not encode universally conserved genes such as the 16S or 18S rRNA genes of prokaryotes and eukaryotes, respectively, and are highly genetically diverse (Carding et al., 2017), which impedes rapid and effective detection of viruses in the GI. Development of novel and improved methods for detection of contamination would substantially facilitate quality control assessment and such methods would find immediate application for use in GF animal models.

### Tips for the Establishment of GF Mouse Colony

Rat and mouse belong to the rodent and they are very similar in many ways. In general, protocols for establishment of GF rat colony are applicable to establish the GF mouse colony. Methods for assembling and sterilizing isolator, importing supplies, performing the hysterectomy, hand feeding the neonates by gavage and assessing the microbiological status by culture, Gram-staining and PCR based on 16S rRNA gene are the same. However, it should be noted that during the hand feeding period, the amount of artificial milk which was given to the suckling GF mouse is less than the rat due to their difference in the body size. The depth of the insertion of the gavage needle is different as well. Moreover, the day of giving soft food and weaning are different too. According to our experience, since mouse are much smaller than the rat, hand feeding of the GF suckling mouse by gavage might be more difficult than the suckling GF rat. Proficiency in gavage operation of experimenters, environmental conditions and the status of the neonates are the main factors to determine whether the experiment is successful or not. Therefore, the survival rate of weaned GF mouse is difficult to predict. Moreover, there is no need to worry too much about the cost of this experiment, the isolator bubble is reusable and other reagents are commonly used.

## Conclusion

We present an easy-to-use protocol for the establishment and maintenance of a GF rat model in an isolated environment. The validity of this protocol was confirmed by evaluation of bacterial contamination testing using three different methods. The most challenging parts in establishing and maintaining a GF rat model is, in our experience, the handfeeding of suckling rats until weaning and maintaining a GF environment. Repeated assessment of the GF status of the model is essential and improved methods for detecting contamination with increased sensitivity and accuracy are needed. GF rat models are ideal for investigating the interaction between the host and its microbiota relating to disease and the protocol presented herein will facilitate the establishment of experimental models aimed for microbiota-related research.

## Data Availability Statement

The raw data supporting the conclusions of this article will be made available by the authors, without undue reservation, to any qualified researcher.

## Ethics Statement

The animal study was reviewed and approved by Laboratory Animal Ethics Committee of the First Affiliated Hospital of Medical College of Zhejiang University.

## Author Contributions

LQ, SM, YL, and JZ performed all the experiments together. LQ and ZY designed the manuscript. LQ drafted the manuscript and prepared all the figures and tables. LQ, ZY, MY, and LL revised the manuscript for publication.

## Conflict of Interest

The authors declare that the research was conducted in the absence of any commercial or financial relationships that could be construed as a potential conflict of interest.
